# Musculoskeletal ultrasound imaging of the plantar forefoot in patients with rheumatoid arthritis: inter-observer agreement between a podiatrist and a radiologist

**DOI:** 10.1186/1757-1146-1-5

**Published:** 2008-07-28

**Authors:** Catherine J Bowen, Keith Dewbury, Madeline Sampson, Sally Sawyer, Jane Burridge, Christopher J Edwards, Nigel K Arden

**Affiliations:** 1 School of Health Professions and Rehabilitation Sciences, University of Southampton, Southampton, UK; 2 Ultrasound Imaging, Department of Radiology, Southampton University Hospitals NHS Trust, Southampton, UK; 3 Department of Rheumatology, Southampton University Hospitals NHS Trust, Southampton, UK; 4 MRC Epidemiology Resource Centre, University of Southampton, Southampton, UK

## Abstract

**Background:**

The use of musculoskeletal ultrasound (MSUS) in the diagnosis and management of foot and ankle musculoskeletal pathology is increasing. Due to the wide use of MSUS and the depth and breadth of training required new proposals advocate tailored learning of the technique to discrete fields of practice. The aims of the study were to evaluate the inter-observer agreement between a MSUS radiologist and a podiatrist, who had completed basic skills training in MSUS, in the MSUS assessment of the forefoot of patients with Rheumatoid Arthritis.

**Methods:**

A consecutive sample of thirty-two patients with rheumatoid arthritis was assessed for presence of synovitis, erosions and bursitis within the forefoot using MSUS. All MSUS assessments were performed independently on the same day by a podiatrist and one of two Consultant Radiologists experienced in MSUS.

**Results:**

Moderate agreement on image acquisition and interpretation was achieved for bursitis (kappa 0.522; p < 0.01) and erosions (kappa 0.636; p < 0.01) and fair agreement for synovitis (kappa 0.216; p < 0.05) during the primary assessments. Following a further training session, substantial agreement (kappa 0.702) between the two investigators was recorded. The sensitivity of the podiatrist using MSUS was 82.4% for detection of bursitis, 83.0% for detection of erosion and 84.0% for detection of synovitis. Specificity of the podiatrist using MSUS was 88.9% for detection of bursitis, 80.7% for detection of erosion and 35.9% for detection of synovitis.

**Conclusion:**

This study demonstrated good inter-observer agreement between a podiatrist and radiologist on MSUS assessment of the forefoot, particularly for bursitis and erosions, in patients with rheumatoid arthritis. There is scope to further evaluate and consider the role of podiatrists in the MSUS imaging of the foot following appropriate training and also in the development of reliable protocols for MSUS assessment of the foot.

## Introduction

As technology has improved, clinical expertise in performing musculoskeletal ultrasound (MSUS) has also advanced dramatically [1,2]. The advantages of ultrasound imaging over conventional radiography, computed tomography, radioisotope scan and MRI (Magnetic Resonance Imaging) are reported as being that it is: painless, does not use ionising radiation, less expensive, can be performed real time, needs no special environment and is clinically readily accessible [1].

There have been a number of studies that have utilised MSUS to diagnose specific soft tissue foot pain in healthy volunteers [3-5] and soft tissue problems in Rheumatoid Arthritis (RA) [6,7]. More specifically for the forefoot ultrasonographic findings of metatarsophalangeal joint (MTPJ) synovitis [8,9], plantar flexor tenosynovitis [10] and plantar bursitis have been described [11].

MSUS is, however, affirmed as being highly operator dependent [12]. The procedure itself has no known specific side effects, although harm that may result from incorrect acquisition and interpretation of images has been highlighted [2]. The reported challenges in training for this skill are primarily due to the quality and interpretation of the ultrasound images that are acknowledged as being greatly dependent on the expertise and experience of the operator [13]. Knowledge about the basic principles relevant to sound waves and a detailed anatomical knowledge of the structures under investigation are therefore mandatory [14].

Models for the use of MSUS by clinicians other than radiologists, have been demonstrated [15,16] and specific competencies for MSUS performed by non radiologists have been rigorously developed [17-19]. Within the most recent proposed framework for development of competencies in MSUS scanning techniques, due to the training challenges, tailored learning to areas directly relevant to a clinician's discrete field of practice is proposed [19].

This study provided an opportunity to evaluate the inter-observer agreement between a radiologist and podiatrist who had followed recommended training guidelines for MSUS, in the MSUS assessment of the forefoot of patients with RA. This work forms part of an ultrasound based research programme investigating the clinical relevance of soft tissue inflammation within the forefoot of patients with RA.

## Materials and methods

### Patient selection

The initial approach for recruitment was based upon potential participants with rheumatoid arthritis diagnosed according to the American College of Rheumatology (ACR) criteria, [20] and starting anti-TNF-α therapy (infliximab, etanercept, adalimumab) all of whom were attending the Southampton University Hospitals NHS Trust Rheumatology Department. Patients were excluded if they had previous surgery to the forefoot, had corticosteroid injection therapy to the forefoot within the previous 3 months prior to commencement of the study; had concomitant musculoskeletal disease (eg, primary osteoarthritis, gout, Pagets disease, systemic lupus erythematosus), had a serious medical or psychological disorder that would affect the study protocol or were unable to give informed consent. Local research ethics committee approval was secured and all patients gave informed written consent prior to their participation.

### Confirmation of RA diagnosis and disease activity

Prior to data collection, the diagnosis of RA was confirmed by the supervising rheumatology consultant using the ACR criteria. Following acceptance into the study all participants were assessed by a trained specialist rheumatology nurse and Disease Activity Scores (DAS28) were calculated [21].

### Data collection

Data collection took place between March 2004 and August 2007. On each occasion, the same treatment bays and ultrasound facilities were utilised in an attempt to standardize environmental factors, i.e. room temperature and scanning positions. All investigators performing MSUS were blinded to the other's results in order to minimize the risk of bias.

### Assessment of demographic and clinical characteristics

General demographic data of age, gender, disease duration, presence of rheumatoid factor, weight and limb dominance were obtained from the clinical notes and rheumatology department database. Clinical characteristics included visual analog scale (VAS 100 mm) assessment for the patient's global impression of health and disease activity and the number of painful, tender and swollen joints calculated as DAS28.

### Imaging Data

**Figure 1:**
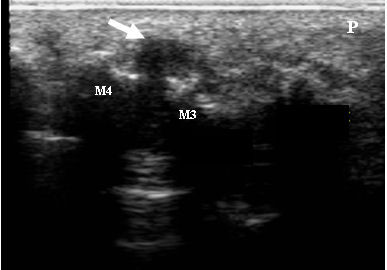
**A MSUS image of the left foot plantar metatarsal area of a study participant with RA**. The MSUS image demonstrates bursitis as a demarcated complex mass protruding beyond the 3rd and 4th metatarsal heads with hypertrophied synovium and anechoic spaces containing synovial fluid (arrow). The image is seen from the plantar aspect and in the transverse plane. M4: 4^th ^metatarsal head; M3: 3^rd ^metatarsal head; P: plantar fat pad.

**Figure 2:**
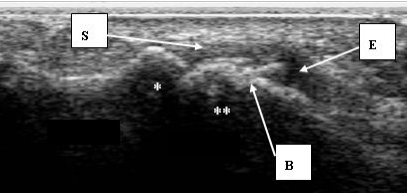
**A MSUS image of the plantar aspect of the right forefoot**. The MSUS image demonstrates synovial thickening, joint effusion and bone changes within the right fifth plantar MTPJ of a study participant with RA. The image is seen from the plantar aspect and in the longitudinal plane *:proximal phalanx; **:metatarsal head, S:synovial thickening; E:joint effusion; B:bone changes.

The presence and location of any bursitis (see Figure 1) across the plantar forefoot region and any synovial thickening/synovitis and erosion (see Figure 2) within the second and fifth MTPJs identified by MSUS was recorded. Thirty minutes was allowed for each assessment and thus was limited to two joints within each foot. The second MTPJ was selected for investigation because it is in line with the centre of load through the forefoot during gait [22], it is easily accessible for the MSUS probes, and was considered representative of the MTPJs. The first MTPJ was excluded due to the sesamoid bones underlying its plantar aspect. The fifth MTPJ was also selected for its ease of accessibility with the MSUS probes and as it has been reported as being the most common site of radiographic and sonographic erosion in RA [23].

### Imaging Equipment

A Diasus diagnostic ultrasound scanner (Dynamic Imaging, Livingston, Scotland UK), was used by the podiatrist. The Diasus scanner operates as a system with dual probe, but for the study, a L5–10 MHz 26 mm ultra wideband linear array probe was used.

A Philips HDI 5000 System was used by the radiologists (Royal Philips Electronics, Netherlands). The Philips system also operates with broadband linear probes and in order for direct comparisons to be made within the study a L5 – 12 MHz probe was used.

### MSUS Scanning Training and Protocol Development

The Podiatrist attended the BSR (British Society for Rheumatology) core MSUS course that is organised by experts in MSUS providing hands on experience at different levels of basic, intermediate and advanced specifically for non-radiologists within the UK [24]. MSUS practice and mentorship in scanning technique from two experienced MSUS Radiologists took place over a further period of six months following the BSR course.

On the same day the MSUS foot scans were performed independently and without access to the clinical findings by the podiatrist and one of two Consultant Radiologists experienced in MSUS (Table 1). Scanning was in B-Mode and recorded according to the EULAR (European League Against Rheumatism) standard approaches (i.e. from the sole of the foot) [14]. Each MTPJ and inter-metatarsal space was scanned longitudinally and transversely from the plantar view.

**Table 1:** Overview of MSUS Scanning Protocol

1.	The nature of the test was explained
2.	The forefeet of all participants were scanned by the investigator using a broadband linear probe of 5 – 10 MHz frequency.
3.	The participant was asked to sit in a supine position on the bed.
4.	The participant's hosiery was removed and the ultrasound probe placed on the plantar aspect of each foot.
5.	Scans were taken according to the EULAR standard approach from the plantar aspect of the foot [14].
6.	Each MTP joint and inter-metatarsal space was scanned longitudinally and transversely from the plantar view.
7.	Images of the plantar aspects of the forefoot in both feet were recorded in transverse and longitudinal aspects and saved on the ultrasound machine hard drive.
8.	Observations of synovitis and erosions of the second and fifth metatarsophalangeal joints and any evidence of bursitis were noted on a separate data collection sheet.

At the end of the data collection period, a consensus meeting took place between one of the radiologists and the podiatrist. During this meeting, all archived study images recorded by the Diasus (Dynamic Imaging Ltd, Livingston, Scotland, UK) ultrasound system were reviewed and discussed and confirmed by the radiologist as being bursitis, synovitis, erosion, or normal.

Following the consensus meeting, thirty six images were randomly selected from the Diasus (Dynamic Imaging Ltd, Livingston, Scotland, UK) ultrasound system archived data by an independent research assistant. All thirty six images were numbered, logged and the sequence for image viewing was randomized in an unrestricted random method of allocation by the research assistant. Both the primary investigator and consultant radiologist were blinded to the image selection procedure.

The two investigators independently scored all 36 images for the presence of bursitis, synovitis, erosion or normal and both were blind to each others findings.

### Statistical analysis

Data evaluation and statistical analysis were performed using SPSS version 14.0 software (SPSS, Chicago IL). The data was initially examined using histograms and scatter plots to identify 'outliers' that may have occurred due to data entry bias or normal biological outliers.

Inter-observer agreements were calculated by overall agreement (percentage of observed exact agreement) and kappa statistics (unweighted for dichotomous scoring, ie. presence or absence of bursitis, synovitis and erosion). Sensitivity and specificity of the podiatrist's results were calculated from cross-tabulation against the radiologist's results as the gold standard.

## Results

Thirty two patients with RA were recruited. One patient withdrew due to time issues during the visit. Thirty one patients completed the study. There were 24 female and 7 male patients, 12 seronegative and 19 seropositive. The mean age was 59.58 (SD 10.1) years and all patients had active disease with mean DAS scores of 5.8 (SD 0.9) (Table 2). Bursitis was present in 51/62 feet, synovitis in 81/124 MTP joints and erosions in 53/124 MTP joints scanned by the Radiologist (Table 3).

**Table 2:** Demographic characteristics of the study participants

Variable	No	Mean (SD)	Range
Age (years)	31	59.58 (10.14)	37–76
Time since RA diagnosis (years)	31	11.32 (10.57)	1–39
Weight (Kg)	31	70.66 (15.35)	47.70–107.50
Overall well being (100 mm VAS)	28	60.29 (21.12)	20–100
Disease activity Score-28 joints	29	5.76 (0.93)	3.91–7.52

* SD = standard deviation; RA = Rheumatoid arthritis; VAS = visual analog score.

**Table 3:** Relation between the podiatrist's and radiologist's results of MSUS scans for the detection of plantar forefoot

	**Pod presence (N)**	**Pod Absence (N)**	**Total (N)**
**Rad Bursitis Presence (N)**	42	9	51
**Rad Bursitis Absence (N)**	1	8	9
**Rad Erosion Presence (N)**	44	9	53
**Rad Erosion Absence (N)**	11	46	57
**Rad Synovitis Presence (N)**	68	13	81
**Rad Synovitis Absence (N)**	25	14	39

bursitis (N = 62 feet), erosion (N = 124 joints) and synovitis (N = 124 joints) in the study participants.

*Pod:Podiatrist; Rad = Radiologist

Overall agreement was 83.3% for presence or absence of bursitis, 81.8% for presence or absence of erosion and 68.3% presence or absence of synovitis. Kappa scores for from the primary data revealed moderate agreement for bursitis (kappa 0.522; p < 0.01) and erosions (kappa 0.636; p < 0.01) and fair agreement for synovitis (kappa 0.216; p < 0.05).

The sensitivity of the podiatrist using MSUS was 82.4% for detection of bursitis, 83.0% for detection of erosion and 84.0% for detection of synovitis. Specificity of the podiatrist using MSUS was 88.9% for detection of bursitis, 80.7% for detection of erosion and 35.9% for detection of synovitis.

Following the consensus meeting and test on a randomised selection of thirty six images from the DIASUS machine, good levels of agreement were achieved between the podiatrist and radiologist for 8/9 bursitis, 4/10 synovitis, 6/9 erosion, 8/8 normal images (kappa 0.702; p < 0.01).

## Discussion

The use of MSUS assessment of the foot and ankle in clinical practice would be beneficial to patients with RA in facilitating more effective timely referral and management of foot problems. This is the first study to investigate tailored learning of MSUS to the discrete field of foot and ankle practice in evaluating the inter-observer agreement in the use of MSUS between an allied health professional (podiatrist) and an expert radiologist on imaging of the foot. We have demonstrated good agreement for bursitis and erosions, but only fair agreement for the presence of synovitis.

Competency assessment in MSUS is an important issue [17]. Usually, reliability in technique is reported by rheumatologists who have trained or are being trained in MSUS that are tested against experienced MSUS sonographers or radiologists [25-28]. The podiatrist in this study had followed recommended BSR training in MSUS techniques followed by further training and mentorship from expert radiologists. Podiatrists are regularly involved in the assessment and management of musculoskeletal foot and ankle pathology. With extended scope practice in the use of MSUS by podiatrists there are the potentially valuable benefits to patients mentioned above, as well as lower costs in service provision.

In our study overall exact agreement between the radiologist and podiatrist was recorded as 83.3% for bursitis, 68.3% for synovitis and 81.8% for erosions. Acceptable sonographic images were obtained by the podiatrist with moderate agreements for bursitis (kappa 0.522, p <0.01) and erosions (kappa 0.636, p <0.01). Low agreements for synovitis (kappa 0.216, p <0.05) were obtained initially, however following further training, levels of agreement for all three variables increased to a good standard (kappa 0.702, p <0.01).

Within the MSUS literature the foot is under-investigated and those who have reported on assessments of the foot joints have observed similar low agreement scores for synovitis [25-28]. Inter-observer reliability among 14 experts in MSUS produced overall good agreements for all examined joints (kappa 0.76) although low agreement for ankle/toe joints (0.28 kappa) was reported [27]. In evaluating scanning technique and diagnostic criteria, a group of 23 experts in MSUS who scanned shoulder, wrist/hand, ankle/foot and knee joints, reported exact overall agreement of 91% for synovitis, 87% for cortical abnormalities and 83.5% for bursitis but only 'fair' agreements for the ankle and foot region (kappa 0.54) [28]. In MSUS examination of synovitis of the metacarpophalangeal joints and MTPJs the learning curve of three rheumatologists in MSUS techniques was investigated [26]. The agreements at the final evaluations were good for two of the fellows, (kappa 0.63 and 0.62), consistent with our findings, however for the third fellow was poor (kappa 0.18). This highlights the variability of learning requirements for the technique [26].

The use of MSUS by the podiatrist within this study did reveal good sensitivity and specificity for detecting forefoot bursitis (82.4% and 88.9%) and erosion (83.0% and 80.7%), although in detection of MTP synovitis sensitivity was good, specificity was low (84.0% and 35.9%) with over-reporting of false positives. As reported by others [27] we did encounter difficulties in using MSUS to detect synovitis in the MTPJs from the plantar aspect of the foot, especially if deformities and/or subluxation of those joints were present. Interestingly others have scanned each joint in the dorsal aspect of the hands and feet, reporting that this technique was preferred to a palmer or plantar scan because of its reliability for detecting synovitis [26] although they did not mention technique where deformity and subluxation existed in the MTPJs. From our experiences within this study we recommend that further work on establishing reliability of protocols for MSUS assessment of the foot and ankle be conducted.

A number of potential limitations within this current study should be acknowledged. The prevalence of synovitis, erosion and bursitis within the forefoot was not validated by any other 'gold standard' imaging technique such as MRI. Other authors have not attempted to address this issue either although results from the OMERACT MSUS special interest group highlighted the need to consider comparison of MSUS data with histology and MRI [29]. MRI however remains more costly and less readily accessible than MSUS which made it less feasible to be included in this study. Limitations relating to the sample should also be highlighted. The sample was relatively small and comprised patients with severe disease therefore generalizibility to the whole population of patients with RA needs to be confirmed. Finally, the images selected for analysis after the consensus meeting were taken from part of the cohort that the podiatrist and radiologist used for the initial reliability analyses. Whilst the consensus meeting did take place once data collection was complete, an element of recall bias may have been introduced and thus should be acknowledged.

## Conclusion

This study was the first to attempt to investigate inter-observer agreement in the use of diagnostic ultrasound between an allied health professional (podiatrist) and an expert radiologist. Performance of MSUS in image acquisition and interpretation by the podiatrist was of an acceptable standard during the primary investigation and following further training levels of agreement increased to a good standard. The foot is a complex structure with many small joints and podiatrists are well placed to be involved in its MSUS assessment, however we have highlighted the importance of appropriate training and further mentorship from experts in MSUS imaging techniques. Finally, we have recommended that further work is undertaken in establishing reliable protocols for MSUS assessment of the foot.

## List of Abbreviations

MSUS: Musculskeletal ultrasound; MRI: Magnetic Resonance Imaging; MTPJ: Metatarso-phalangeal joint; RA: Rheumatoid Arthritis; ACR: American College of Rheumatology; TNF: Tumour necrosis factor; DAS28: 28 joint Disease Activity Score; VAS: Visual Analog Scale; BSR: British Society for Rheumatology; RCR: Royal College of Radiologists; EULAR: European League Against Rheumatism; SD: Standard Deviation; OMERACT: Outcome Measures in Rheumatology Clinical Trials.

## Competing interests

No benefits in any form have been received or will be received from a commercial party related directly or indirectly to the subject of this article.

The authors declare that they have no competing interests.

## Authors' contributions

CB conceived of the study and participated in its design, coordination and collection of clinical variables, MSUS assessments and writing of the manuscript. KD participated in the design of the study, MSUS assessments, consensus meeting and helped to draft the manuscript. MS participated in the MSUS assessments, and helped to draft the manuscript. SS participated in study design and coordination. JB participated in the design of the study and helped to draft the manuscript. CE participated in the design of the study, coordination, confirmation of RA diagnosis and helped to draft the manuscript. NA participated in the design of the study, coordination and helped to draft the manuscript. *All authors read and approved the final manuscript*
